# Antidyskinetic Treatment with MTEP Affects Multiple Molecular Pathways in the Parkinsonian Striatum

**DOI:** 10.1155/2017/5798734

**Published:** 2017-10-30

**Authors:** Jing-ya Lin, Zhen-guo Liu, Cheng-long Xie, Lu Song, Ai-juan Yan

**Affiliations:** Department of Neurology, Xin Hua Hospital Affiliated to Shanghai Jiao Tong University School of Medicine, 1665 Kongjiang Road, Shanghai 200092, China

## Abstract

Parkinson's disease is characterized by dopaminergic neuron loss and dopamine (DA) depletion in the striatum. Standard treatment is still focused on the restoration of dopamine with exogenous L-Dopa, which however causes L-Dopa-induced dyskinesia (LID). Several studies have shown that antagonism of the metabotropic glutamate receptor 5 alleviates LID, but the underlying mechanisms have remained unclear. We set out to determine where this alleviation may depend on restoring the equilibrium between the two main striatofugal pathways. For this purpose, we examined molecular markers of direct and indirect pathway involvement (prodynorphin and proenkephalin, resp.) in a rat model of LID treated with the mGluR5 antagonist MTEP. Our results show that MTEP cotreatment significantly attenuates the upregulation of prodynorphin mRNA induced by L-Dopa while also decreasing the expression levels of proenkephalin mRNA. We also examined markers of the mGluR5-related PKC/MEK/ERK1/2 signaling pathway, finding that both the expression of PKC epsilon and the phosphorylation of MEK and ERK1/2 had decreased significantly in the MTEP-treated group. Taken together, our results show that pharmacological antagonism of mGluR5 normalizes several abnormal molecular responses in the striatum in this experimental model of LID.

## 1. Introduction

The loss of dopaminergic neurons in the substantia nigra and the depletion of dopamine are main neuropathological features of Parkinson's disease (PD) [1, 2]. Current standard treatment for PD still focuses on the dopamine replacement therapy with L-Dopa [2]. However, long-term use of this drug causes a decrease in the efficacy and disabling abnormal involuntary movement (AIM), which was known as L-Dopa-induced dyskinesia (LID) [3].

The striatum is deeply involved in handling the motor information that comes from the cortex. In the classic model of striatal connectivity, there are direct and indirect pathways in the striatum [4]. Both direct and indirect pathways originate from medium spiny efferent neurons (MSNs). In the indirect pathway, the MSNs express enkephalin, while in the direct pathway the MSNs express dynorphin [5]. The first report of a direct correlation between prodynorphin level and LID was provided by Cenci et al. [6]; furthermore, they also proposed that an imbalance between the direct and indirect pathways leads to the appearance of the motor signs of parkinsonism [7]. In a recent research, it was also reported that both the dynorphin and enkephalin expression levels were involved in the development of motor complications while being treated with L-Dopa. Sgroi et al. pointed out that, on the one hand, the preproenkephalin level was increased before the use of L-Dopa after 6-OHDA lesion, and it remained high after L-Dopa washout; on the other hand, there is a correlation between the rotational AIM and preproenkephalin level in the on state [8]. All these phenomenons suggested that the increased proenkephalin mRNA level may be a prerequisite to the locomotor sensitization before L-Dopa treatment [8]. On the other hand, the association of prodynorphin with LID is clearer than that with proenkephalin, because it has been consistently proven in several studies [5, 8–10].

We also know that overactivation of glutamatergic signaling and the hypersensitivity of the glutamatergic system in the basal ganglia play an important role in the pathophysiology of LID. Several groups of researchers reported that the blockade of the metabotropic glutamate receptor 5 (mGluR5) could attenuate the LID [11–16]; emerging evidence has come to support the important role of mGluR5 in the development of LID [17]. But the mechanism behind the alleviation effect is unclear [18]. It is of great importance to investigate the extent to which the blockade of the metabotropic receptor 5 affects the imbalance between direct and indirect pathways and to investigate what the molecular alterations in the mGluR5-related signaling pathway are, in order to interpret the antidyskinesia effect of the antagonists of mGluR5. In order to address these questions, we tested the protein level of protein kinase C (PKC), MEK, and extracellular signal-regulated kinase 1/2 (ERK1/2) in the mGluR5 mediated PKC/MEK/ERK1/2 signaling pathway; we also tested the mRNA expression level of prodynorphin (PDyn) and proenkephalin (PEnk) in order to verify the effect of the blockade of mGluR5 on the direct and indirect pathways.

## 2. Materials and Methods

### 2.1. Experimental Design

**Figure 1:**
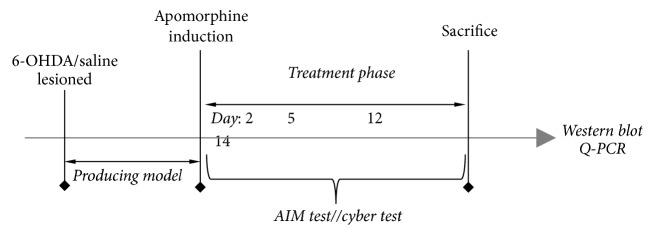
The protocol of the experiment. Dopamine depletion was induced by 6-OHDA injections in the medial forebrain bundle (MFB) while the sham group was injected with saline in the MFB. Three weeks later, the rats that exhibited apomorphine-induced rotations exceeding 7 turns/min in the apomorphine induction test were put to the subsequent experiment. The sham and PD groups were injected with saline once daily; the LID group received L-Dopa (25 mg/kg) and benserazide (6.25 mg/kg) cocktail once daily, while the MTEP group received MTEP (5 mg/kg) 30 min before the injection of L-Dopa and benserazide. All treatments were performed for 14 days; behavior tests like AIM test, open field test, and cylinder test were performed on days 2, 5, 12, and 14. Two hours after the last injection on the 14th day, all groups were sacrificed for western blot and Q-PCR.

As shown in Figure 1, Sprague-Dawley rats were given 6-OHDA injections in the medial forebrain bundle (MFB) in the right side of the brain, while five SD rats were given saline injections instead as sham group. Contralateral turning behavior was tested on the 6-OHDA rats after the apomorphine injection. The rats whose apomorphine-induced rotations are more than 7 turns/min were enrolled in the follow-up experiments as the Parkinson disease model animals. The selected rats were distributed into 3 groups randomly. The first group was given L-Dopa (25 mg/kg, i.p.) plus benserazide (6.25 mg/kg, i.p.) once daily for 14 days, labeled as LID group; the second group was given saline once daily for 14 days, labeled as PD group; and the third group was given MTEP (5 mg/kg, i.p.) 20 mins before the injection of L-Dopa plus benserazide for 14 days, labeled as MTEP group. During this period, AIM and open field tests were conducted in all the groups on days 2, 5, 8, 12, and 14 by a new assigned observer who did not know the details of each group. The animals were sacrificed 2 h after the last injection for western blot and Q-PCR.

### 2.2. Animals

The study was conducted on adult female Sprague-Dawley rats (Sprague-Dawley, 180–220 g, Sippr-BK Ltd., Shanghai, China). The maintenance of the animals followed the guidelines of the National Institutes of Health for the care and use of laboratory animals. All experimental protocols involving animals were approved by the Ethical Committee of the Medical School of Shanghai Jiaotong University.

### 2.3. Drugs

L-Dopa and benserazide were purchased from Sigma-Aldrich (Spain), and MTEP was purchased from Abcam (UK). All drugs were freshly prepared in 0.9% saline before use. L-Dopa (Sigma-Aldrich) plus benserazide (Sigma-Aldrich) was administrated once daily. MTEP (3-[(2-methyl-1,3-thiazol-4-yl)ethynyl]-pyridine, Abcam, UK) preceded the L-Dopa cocktail 20 minutes earlier once daily for 2 weeks.

### 2.4. 6-OHDA Lesions and Treatment

For the stereotaxic procedure, the rats (weighing 180~220 g) were anesthetized with 10% chloral hydrate (0.5 ml/100 g) deeply. As previously described [19, 20], the surgery was performed on the right side medial forebrain bundle (MFB) by unilateral injection of 6-OHDA (20 mmol/L, containing 0.02% ascorbic acid; Sigma-Aldrich, Spain) at the coordination of MFB. Sham-operated rats received the vehicle at the same spot. A volume of 4 *μ*l was injected in each spot. 21 days later, all the rats were tested with 0.05 mg/kg subcutaneous injection of apomorphine (i.p. WOKO, Japan). Contralateral rotation test was performed and the animals exhibiting full body turns of over 7 turns/min towards the unlesioned side were enrolled and started on a 2-week course of daily i.p. injections of MTEP (5 mg/kg) followed by L-Dopa (25 mg/kg) plus benserazide (6.25 mg/kg) 20 min later.

### 2.5. Behavior Assessment

To evaluate LID, we used the combined “time *∗* amplitude” scale which was first applied by Rylander et al. [21]; mice were observed in a clear-glass cylinder and were observed and evaluated by a trained experimenter. Rat abnormal involuntary movements (AIMs) were classified into three subtypes: axial, limb, and orolingual dyskinesia. Each individual dyskinesia subtype scores from 0 to 4. During a period of 120 min following levodopa injection, the severity of AIM was assessed at a 20 min interval (20, 40, 60, 80, 100, and 120 min). The ALO AIM scores were rated at 2, 5, 12, and 14 days during levodopa treatment. Motor coordination was evaluated with the cylinder test at 2, 5, 12, and 14 days and the locomotor activities were tested by the open field test on the 2nd and 14th days during levodopa treatment. The open field test and cylinder test were the index of Parkinsonian disability. In the cylinder test, the rats were placed in a glass cylinder with a diameter of 22 cm and a height of 35 cm to record forelimb use during vertical exploration for 60 min. During a period of 60 min before levodopa treatment, the forelimb functional test was assessed every 15 min (3 min monitoring period for each). The final value was expressed in terms of the percentage use of the impaired forelimb compared with the total number of limb use movements. All the behavioral experiments were carried out with the observer blinded to the groups and treatment.

### 2.6. Western Blot

Striatum tissue of rats was harvested 2 hours after the last injection of L-Dopa and homogenized in RIPA lysis buffer (Beyotime Institute of Biotechnology) and fresh-added protease inhibitor cocktail and phosphatase inhibitor (Roche Diagnostics, Switzerland). And then the cytosol was prepared by centrifugation at 12000*g* for 10 min at 4°C. An equal amount of protein (40 ug) from each sample was added to 10% SDS-PAGE and separated by electrophoresis and transferred to polyvinylidene difluoride membranes in a Tris-glycine transfer buffer. Each sample was heated at 95°C previously for 5 min. The membrane was blocked for half an hour at room temperature (26°C) in 5% instant nonfat milk and then incubated with primary antibodies corresponding to epsilon PKC (1 : 1000, Abcam, UK), p-MEK and MEK (1 : 1000, Abcam, UK), p-ERK1/2 and ERK1/2 (1 : 1000, Cell Signaling Technology (CST), USA), and *β*-actin IgG (diluted 1 : 1000; Beyotime Institute of Biotechnology), respectively, at 4°C overnight (14–16 hours). The membranes were subsequently washed with TBST (50 mM Tris-HCl (pH 7.5), 150 mM NaCl, and 0.05% Tween 20) and then incubated with horseradish peroxidase conjugated secondary anti-rabbit and anti-mouse IgG (diluted 1 : 1000; Beyotime Institute of Biotechnology) for one hour at room temperature. The signal was visualized by ECL (A : B = 1 : 1; Millipore) and quantified using Quantity One software (Image Lab). All individual protein bands were compared with their internal control actin values in order to provide relative protein abundance. All the procedures were repeated 3 times.

### 2.7. Real-Time PCR

Striatal tissues of rats were homogenized and total ribonucleic acid (RNA) was extracted by TRIzol reagent (Invitrogen, USA). cDNA was generated from total RNA samples using the Revert Aid First Strand cDNA Synthesis Kit (Takara, Japan). Q-PCR was performed using the ABI 7500 Real-Time PCR System (Life Technologies, USA) according to the supplier's instructions. The primer sequences used in this study were as follows:

  5-CTTGTGTTCCCTGTGTGCAGTG-3 (forward)
  3-AGCAACCTCATTCTCCAAGTCA-5 (reverse) for PDyn mRNA
  5-GAAGATGGATGAGCTTTACCCC-3 (forward)
  3-CAAGGTGTCTCCCTCATCTGC-5 (reverse) for proenkephalin mRNA

Amplification was performed with 40 cycles of denaturation at 95°C for 15 s, annealing at 60°C for 60 s, and extension at 75°C for 20 s using the ABI 7300 Real-Time PCR System (Applied Biosystems, CA, USA). Results were expressed as relative expression corrected to the GAPDH gene. The detector used in real-time PCR reaction is SYBR Green.

### 2.8. Statistical Analysis

Data were expressed as the mean ± standard deviation (SD) unless stated otherwise. Behavioral data were analyzed using Kruskal-Wallis test followed by Dunn's test for multiple comparisons in the case of comparing data over multiple days, or a Mann–Whitney *U* test. The western blot and Q-PCR conformed to normal distribution, and analyses of their data were performed using one-way analysis of variance (ANOVA) followed by LSD post hoc comparisons when appropriate as indicated in the figure legends. *p* values < 0.05 were considered statistically significant. Analysis was performed with GraphPad Prism 5.

## 3. Result

### 3.1. MTEP Prevented the Development of L-Dopa-Induced Dyskinesia

**Figure 2:**
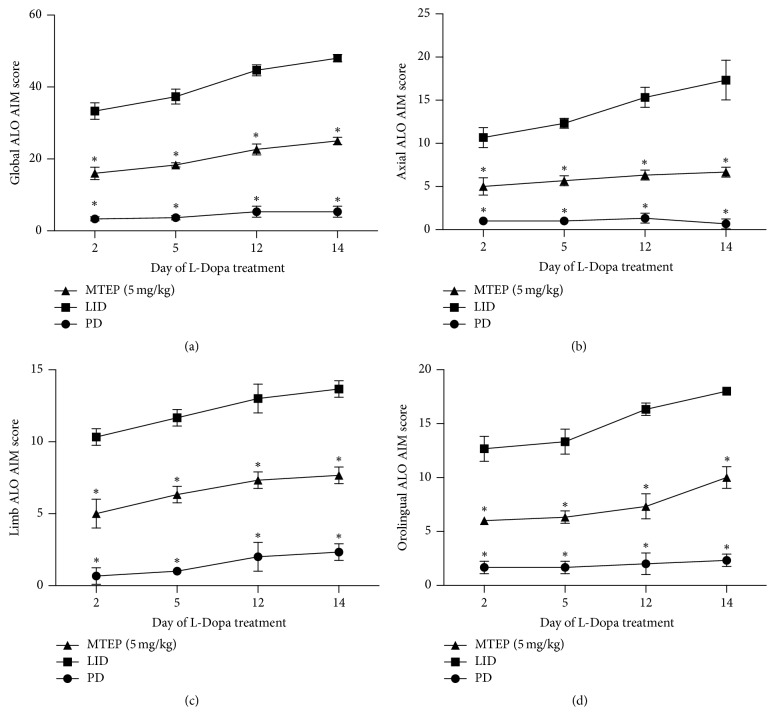
Effect of MTEP on the AIM scores. 14 days' use of MTEP significantly reduced AIM scores. At the 2nd, 5th, 12th, and 14th days, a total AIM score was calculated as the sum of the basic scores multiplied by the amplitude of the score for each AIM subtype: limb, orolingual, and axial, excluding the rotation subtype. (a) Time course of the total scores; sum of the axial, limb, and orolingual subtype scores; (b) time course of changes in the axial scores; (c) time course of changes in the limb score; and (d) time course of changes in the orolingual score. In each testing session, the AIM scores were rated following the administration of the drugs. Data are presented as the mean ± SD. ^*∗*^*p* < 0.01 versus the LID group (Kruskal-Wallis test followed by Dunn's test for multiple comparisons or Mann–Whitney *U* test).

The PD group received saline injection over 14 days, while the LID group was injected with L-Dopa (25 mg/kg) plus benserazide (6.25 mg/kg). The MTEP group was given MTEP (5 mg/kg, i.p.) 20 min before the L-Dopa cocktail injection. The evaluation of the AIM scores included 3 subtypes: axial, limb, and orolingual AIMs. The scores demonstrated a rat dyskinesia scale. We found that 2 weeks of L-Dopa treatment induced full development of LID features, as demonstrated by the increased ALO AIM scores in the LID rats (*p* < 0.05 for treatment effect, *p* < 0.05 for time effect, and *p* < 0.01 for treatment and time interaction, Figure 2). This result is in line with our previous study. AIM scores decreased in the MTEP group. MTEP treatment for 14 days significantly reduced the total dyskinesia scores while the rats of PD group that received saline for 14 days did not develop dyskinesia. Furthermore, the MTEP group demonstrated a reduction in all testing sessions. These data indicate that treatment with MTEP significantly inhibited the development of LID.

### 3.2. MTEP Did Not Compromise the Anti-Parkinsonian Effect of L-Dopa

**Figure 3:**
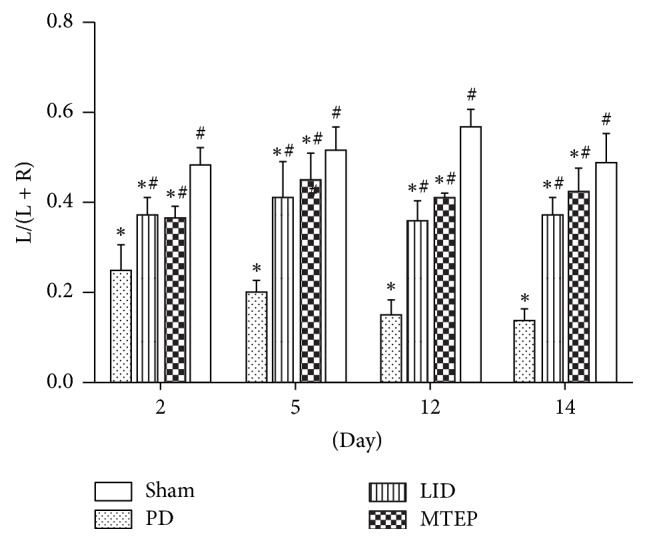
Spontaneous forelimb use of the rat in various experimental groups. Cylinder test. *∗* indicates a significant decrease relative to the sham group (^*∗*^*p* < 0.01), and # indicates a significant increase from PD group (^#^*p* < 0.05). Columns indicate the mean, and bars indicate the SD; the cylinder test was performed on the 2nd, 5th, 12th, and 14th days, using one-way ANOVA followed by Bonferroni post hoc tests.

We then sought to determine whether the administration of antagonists of mGluR5 ameliorated LID compromised the therapeutic response to L-Dopa in PD rats. The cylinder test was used to assess spontaneous forelimb use. We conducted the cylinder test on the 2nd, 5th, 12th, and 14th days. We observed that 6-OHDA-lesioned rats treated with L-Dopa prefer to use the contralateral forelimb to touch the inner wall of the cylinder compared with the 6-OHDA-lesioned rats treated with saline, but the preferential use of the contralateral forelimb was lower compared with the sham group rats (Figure 3, ^*∗*^*p* < 0.01). Data showed that the coinjection of MTEP with L-Dopa did not impact the preferential use of the contralateral forelimb (Figure 3, ^#^*p* < 0.05). There is no significant difference between the LID group and the MTEP group.

### 3.3. Blockade of mGluR5 Prevents the Expression of PKC and Phosphorylation of MEK and ERK1/2 Protein Level

**Figure 4:**
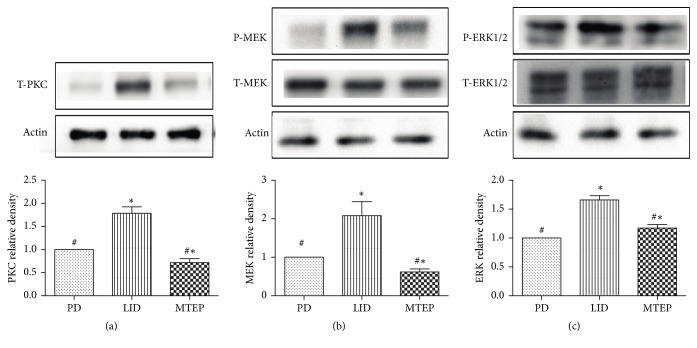
(a–c) Protein levels were evaluated by western blotting of samples from the lesioned side of the striatum (% of PD). (a) Protein level of PKC expressed relative to the level of *β*-actin in the 6-OHDA-lesioned rats treated with saline, L-Dopa (25 mg/kg) plus benserazide (6.25/kg), and MTEP. (b) Protein level of p-MEK level expressed relative to the total MEK level in the 6-OHDA-lesioned rats treated with saline, L-Dopa (25 mg/kg) plus benserazide (6.25/kg), and MTEP expressed relative to the level of *β*-actin in the sample. (c) p-ERK1/2 level expressed relative to the total ERK1/2 level in the 6-OHDA-lesioned rats treated with vehicle, L-Dopa (25 mg/kg) plus benserazide (6.25/kg), and MTEP expressed relative to the level of *β*-actin in the sample. Comparisons with the LID group revealed that MTEP prevented the increase of PKC, p-MEK, and p-ERK1/2 after chronic L-Dopa treatment (^#^*p* < 0.05). Comparisons with the PD group revealed that PKC, p-MEK, and p-ERK levels were increased in the LID group, but there is a minor decrease in the PKC and MEK expression level (^*∗*^*p* < 0.05); there is no significant difference in the p-ERK1/2 expression level. The data represent the mean relative optical density ± SD (one-way ANOVA, *n* = 4 per group).

Protein kinase C was reported to contribute to the development of LID; in particular, the expression level of the novel PKC isoform, PKC epsilon, ipsilateral to the lesion side of the striatum, was increased after chronic L-Dopa treatment [22]. Here, we confirmed that intermittent administration of L-Dopa in hemi-Parkinsonian animals greatly increased the expression level of epsilon PKC, but this enhancement was reversed by the injection of MTEP (^#^*p* < 0.05, Figure 4(a)) compared with the LID group. It was documented that L-Dopa produces pronounced activation of ERK1/2 signaling in the dopamine-denervated striatum through a D1-receptor-dependent mechanism. This effect is associated with the development of dyskinesia [23]. In this study, we found that this elevation in p-MEK and p-ERK1/2 level was reduced in the MTEP group (^#^*p* < 0.05, Figures 4(b) and 4(c)) compared with the LID group. The MTEP group also showed a minor but significant reduction compared with PD in the PKC expression level and phosphorylation of MEK (^*∗*^*p* < 0.05, Figures 4(b) and 4(c)), but there is no significant difference in the phosphorylation level of ERK1/2 between the PD and the MTEP groups (*p* > 0.05).

### 3.4. MTEP Reduces the Expression of Prodynorphin and Proenkephalin in Parkinsonian Rats with LID

**Figure 5:**
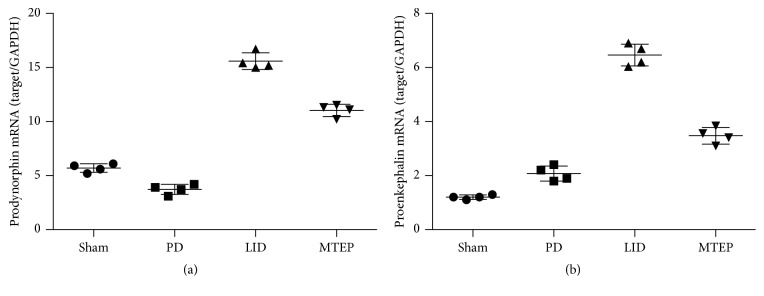
mRNA level of prodynorphin (a) and proenkephalin (b). MTEP reduced prodynorphin and proenkephalin mRNA levels in the striatum of dyskinetic rats. Striatal prodynorphin and proenkephalin mRNA expression levels were determined by real-time PCR. Increased levels of the two genes were found in LID rats. There is a minor but significant (*p* < 0.05) drop in the prodynorphin in the PD group. The antagonist of mGluR5 decreased the mRNA expression level in both prodynorphin and proenkephalin (one-way ANOVA, *n* = 4 per group).

The mRNA expression levels of prodynorphin and proenkephalin were measured by real-time PCR. We found that prodynorphin (Figure 5(a)) expression has a minor reduction in the PD group, but it increased in the LID group; however, MTEP treatment significantly reversed the tendency. The proenkephalin (Figure 5(b)) levels were increased in PD rats primed with L-Dopa. However, MTEP significantly reduced the expression level of proenkephalin in PD rats primed with L-Dopa.

## 4. Discussion

Hypersensitivity and overactivation of glutamatergic signaling in the basal ganglia play a key role in the development of LID [24]. We used a hemi-Parkinsonian rat model of dyskinesia based on unilateral 6-OHDA striatal injection in the MFB, followed with the intraperitoneal administration of chronic L-Dopa daily at a dose of 25 mg/kg in the LID group and saline in the PD group and the injection of MTEP 20 min before the L-Dopa cocktail in the MTEP group. In accord with our previous work [20, 25, 26], these 6-OHDA-lesioned Parkinsonian rats developed progressive dyskinesia following the chronic use of L-Dopa (Figures 2(a)–2(d)). We also confirmed that antagonizing mGluR5 reduced the AIM scores in the rat model animals without ablating the anti-Parkinsonian effect of L-Dopa (Figure 3). In this present study, we explored the possible mechanism of the alleviation effect mediated by the antagonist of mGluR5. We found that PKC level increased in the LID group, which was consistent with the findings of Smith et al.; they also found that the antagonization of PKC could reduce the motor symptoms of LID [22]. We also come up with the result that the phosphorylation of ERK1/2 and MEK was reduced in the MTEP group (Figures 4(a)–4(c)).

Numerous researches had already shown that the PKA signaling pathway in the striatum is closely related to the activation of D1R, which was deeply involved in the expression of LID [19, 20, 27]. After the enhancement of PKA signaling, many downstream molecules like ERK1/2 were upregulated. The phosphorylation of ERK1/2 was also closely correlated with the appearance of L-Dopa-induced dyskinesia [27–29].

It was well documented that there is a close functional interaction between the D1R and the mGluR5; with the long-term use of L-Dopa, the PKC/MEK/ERK1/2 pathway was activated [30], while blockade of the mGluR5 might call back the overactivated signal pathways.

It is well established that the opioidergic neuropeptides dynorphin and enkephalin are also involved in the striatal control of motor and behavioral function [8]. Changes in the striatal expression of proenkephalin and prodynorphin mRNA level have been reported in Parkinsonian rats with L-Dopa-induced dyskinesia. It is known that increased PDyn mRNA is attributed to increased activity in the direct pathway; the expression level of PDyn mRNA has also been reported to be closely related to the genesis of LID [31]. Enkephalin is an important striatal marker in the indirect pathway. In the classic model of the basal ganglia circuit, the enhancement of activities in the direct pathway increases the expression level of prodynorphin mRNA, and the inhibition of the indirect pathway was also reinforced [8, 32]. All these molecular changes lead to further asymmetrical pathological changes and further asymmetrical dysfunction of the neural circuit in the basal ganglia. To our knowledge, it is now established that 6-OHDA-lesioned Parkinsonian rats have an abnormal increase in the mRNA level of the direct (prodynorphin) and indirect (proenkephalin) markers [5, 7, 8]; in this present study, we found that striatal mRNA of proenkephalin was increased in PD rats and continued to increase after intermittent use of L-Dopa; as for the mRNA level of prodynorphin, it exhibited a minor drop in 6-OHDA-lesioned PD rats and this tendency reversed (increased) significantly after priming with L-Dopa in the following days (Figures 5(a) and 5(b)); these were consistent with the findings of Sgroi et al. [8]. After the use of the antagonist of mGluR5, as is shown in Figure 2, coadministration of MTEP (5 mg/kg, i.p.) with L-Dopa (25 mg/kg, i.p.) did not develop severe dyskinesia over the 14-day treatment; the antagonization of the mGluR5 downregulated the overactivated phosphorylation of the ERK1/2 via PKC/MEK/ERK1/2 pathways. On the other hand, the overactivation of the PKA pathway induced the enhancement in both prodynorphin and proenkephalin mRNA expression level after L-Dopa priming. In this study, we demonstrated that, with the antagonizing of the mGluR5, the L-Dopa induced an increase of the expression level of the phosphorylation of ERK1/2, and the mRNA expression levels of prodynorphin and proenkephalin were reduced in the 6-OHDA lesioned rats. On the one hand, the downregulation of both prodynorphin and proenkephalin helps to restore the imbalance of the basal ganglia circuit in the direct and indirect pathways. On the other hand, according to the findings of Sgroi et al., the positive correlation between proenkephalin mRNA in off phase and the rotation AIMs in the on phase indicated that the improved enkephalin level probably represents a prerequisite for locomotor sensitization to subsequent L-Dopa treatment [8]. The alleviation effect on proenkephalin mRNA level mediated by MTEP might reduce this hypersensitivity and contribute to the simultaneous reduction in AIM scores. The result we presented here seemed to have a contradiction with part of the figures provided by Mela et al. [15]; in their paper, they found that the acute injection of L-Dopa together with MTEP did not modify the upregulation of the proenkephalin mRNA induced by DA degeneration, but Mela et al. also confirmed in that very paper that, during the chronic L-Dopa treatment, the mGluR5 antagonism partially blocked the additional upregulation of both prodynorphin and proenkephalin, which was consistent with our presented data. Furthermore, on the one hand, it was reported that the DA can exert its effect on the D2 receptor through a non-cAMP-dependent way [33]; it upregulated the phosphorylation of Akt/GSK3*ββ* pathway to affect the DA-dependent behavior; on the other hand, recent research shows that the antagonism of mGluR5 could inhibit the phosphorylation of Akt/GSK3*ββ* [34]; this might contribute to the restoration of the activity of the indirect pathway and might be followed by a decrease of PPE.

## 5. Conclusion

Hypersensitivity and overactivation of glutamatergic signaling in the basal ganglia play a key role in the development of LID. Antagonizing mGluR5 could reduce the AIM scores in the rodent and primate PD model animals. While the antagonist downregulates the signaling on the PKC/MEK/ERK1/2 pathways, it also reduced the expression level of prodynorphin and proenkephalin mRNA significantly. Antidyskinetic treatment with MTEP affects multiple molecular pathways in the Parkinsonian striatum.
